# Reduce the Sensitivity of CL-20 by Improving Thermal Conductivity Through Carbon Nanomaterials

**DOI:** 10.1186/s11671-018-2496-3

**Published:** 2018-03-27

**Authors:** Shuang Wang, Chongwei An, Jingyu Wang, Baoyun Ye

**Affiliations:** 1 School of Environment and Safety Engineering, North University of China, Taiyuan, 030051 Shanxi China; 2 Shanxi Engineering Technology Research Center for Ultrafine Powder, North University of China, Taiyuan, 030051 China

**Keywords:** Graphene (rGO), Carbon nanotube (CNT), CL-20, Thermal conductivity

## Abstract

The graphene (rGO) and carbon nanotube (CNT) were adopted to enhance the thermal conductivity of CL-20-based composites as conductive fillers. The microstructure features were characterized using scanning electron microscopy (SEM) and X-ray diffraction (XRD), and tested the properties by differential scanning calorimeter (DSC), static electricity accumulation, special height, thermal conductivity, and detonation velocity. The results showed that the mixture of rGO and CNT had better effect in thermal conductivity than rGO or CNT alone under the same loading (1 wt%) and it formed a three-dimensional heat-conducting network structure to improve the heat property of the system. Besides, the linear fit proved that the thermal conductivity of the CL-20-based composites were negatively correlated with the impact sensitivity, which also explained that the impact sensitivity was significantly reduced after the thermal conductivity increased and the explosive still maintained better energy.

**Electronic supplementary material:**

The online version of this article (10.1186/s11671-018-2496-3) contains supplementary material, which is available to authorized users.

## Background

CL-20 (2,4,6,8,10,12-hexanitro-2,4,6,8,10,12-hexaazaisowurtzitane)-based composites could possibly replace various explosive compounds such as RDX and HMX to produce high-performance explosives because of its excellent properties of density and energy. However, it cannot be quickly transmitted after experiencing rapid high-low temperature changes since its poor thermal property, which is easy to form the “hot spot” and seriously endanger the safety and reliability of the weapon system [1–7]. Therefore, it is of great significance to effectively improve the thermal conductivity and reduce the impact sensitivity.

In CL-20-based composites, polymer coating plays an efficient and economical role in enhancing the mechanical and thermal resistance of explosive crystals, and graphite is a helpful ingredient used in composites [5, 6]. Now, it has reached a consensus in increasing the thermal conductivity of polymer composites by adding thermal conducting fillers, especially carbon-based nanomaterials with high thermal conductivity. He et al. used two-dimensional graphene nanoplatelets (GNPs) and carbon nanotubes (CNTs) to enhance the thermal conductivity of PBX, and it was found that the thermal property was excellent with GNPs content of 1 wt% [7–9]; Nika et al. proposed a simple model of graphene lattice thermal conductivity under Klmens framework and found that the thermal conductivity increased with the increasing linear dimensions of graphene flakes [10]; Lee et al. improved the thermal stability of the epoxy resin by fluorinating surface modification of CNT and GNP and mixing them to form a network structure, and this synergism can improve the interfacial bonding with the dispersion [11]; Yu et al. found that there is a synergistic effect between GNPs and SWNT in enhancing the thermal conductivity of the epoxy resin composites [12]; and Li et al. also introduced this synergism of CNTs and GNPs could reduce the CFRP surface resistivity by four orders of magnitude and increased the thermal conductivity by more than seven times [13].

Graphene has a large *π*-conjugated two-dimensional structure with a large phonon mean free path and high electron mobility, providing a large contact area and providing a two-dimensional path for phonon transport [14]. However, the van der Waals’ force between the graphene layers lead to a large interlayer thermal resistance, so that the thermal conductivity perpendicular to the plane direction is significantly lower than the in-plane thermal conductivity, and the distribution of the rGO is intricate and sometimes difficult to form the conduction path on same plane [15]. As a one-dimensional material with tubular structure, the high thermal conductivity and high aspect ratio of CNT is beneficial to improve the heat transfer of polymer composites, and the most important is that CNT could provide more paths for the phonon transport and bridge the rGO and explosives [16]. Therefore, it is considered to combine rGO with CNT to increase the interface with the polymer matrix while reducing the thermal interface resistance, bridging adjacent rGO with one-dimensional CNT to form a three-dimensional thermal conductivity network in order to enhance the heat transfer performance of composite materials [8].

Therefore, in this study, rGO and CNT will be used as fillers in CL-20-based composites together to improve the low thermal conductivity and investigated by SEM, XRD, DSC, et al. Furthermore, the heat transfer mechanism and relationship between thermal conductivity and impact sensitivity are further illustrated.

## Methods

### Synthesis of Nanoscale CL-20/Carbon Material Composites

**Fig. 1:**
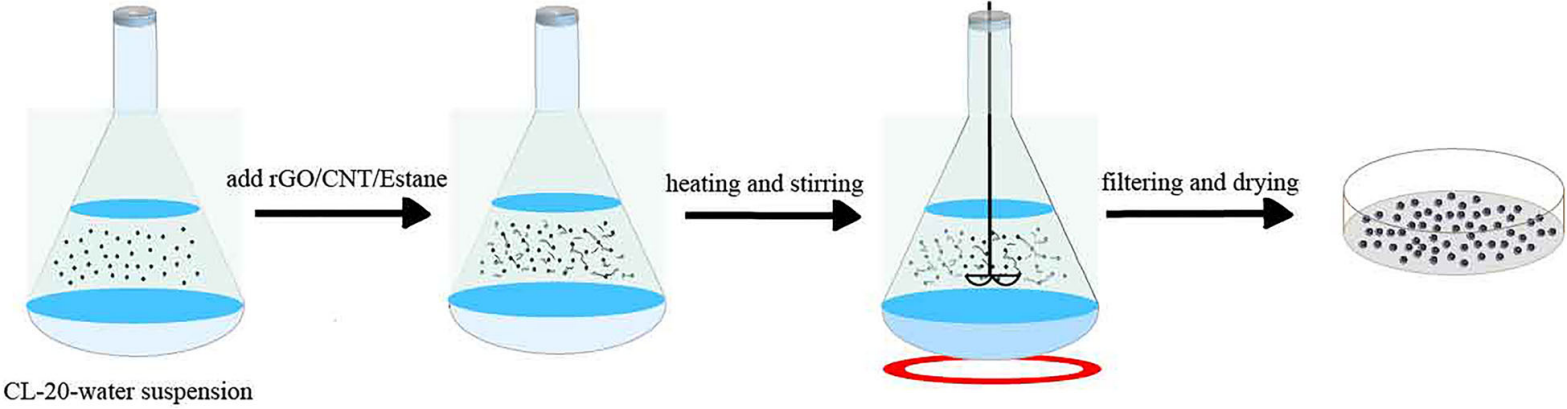
Experimental diagram of CL-20-based composites prepared by water suspension method

CL-20-based composites were prepared by using water suspension method [17, 18], and the specific experimental processes were shown in Fig. 1. Firstly, Estane (purchased from Lu Borun Specialty Chemical Manufacturing Company Ltd.) was added to 1, 2-dichloroethane (obtained from Shun Long Chemical Company Ltd.) to form a solution at a concentration of 3 wt%. Meanwhile, carbon materials [rGO, CNT, or rGO + CNT (rGO, CNT, and the mixture of them (rGO:CNT = 2:1, SWCNT) were provided by Jiangsu Hengqiu Graphite Technology Company Ltd.)] were uniformly dispersed in the estane solution by ultrasonic. Secondly, 20 g milled CL-20 (the raw CL-20 was provided by Liaoning Qingyang Chemical Industry Ltd. and preparation of milled CL-20 was shown in the Additional file 1) was added into 200 ml de-ionized with magnetic stirring to obtain CL-20 suspension. Then, the mixture binder solution was slowly injected into CL-20 suspension and heated in a constant temperature water bath with 70 °C and stirred under pressure at 0.02 MPa until the solvent completely removed. Finally, after cooling, filtering, washing, and evaporation in a vacuum, the CL-20-based composites were obtained. In order to tell the samples apart, the samples were donated as CL-20estane (sample 1), CL-20/rGO (sample 2), CL-20/CNT (sample 3), and CL-20/rGO + CNT (sample 4), respectively.

### Characterization

The surface morphology, mean size, and size distribution of the prepared samples were characterized using scanning electronic microscopy (SEM; SU-8020, Hitachi, Japan). A DX-2700 X-ray diffractometer (Dan Dong Hao Yuan Corporation, Liaoning, China) was used to analyze the element content of CL-20-based composites at a voltage of 40 kV and a current of 30 mA using Cu-Kα radiation.

The samples were analyzed using the DSC-131 differential scanning calorimeter (France Setaram Corporation, Shanghai, China). The conditions of DSC were as follows: sample mass, 0.5 mg; heating rate, 5, 10, 15, 20 K/min; and nitrogen atmosphere, 30 mL/min. The quantitative sample was put into a certain length and inclination of the chute and generated the static charge by friction, the charged sample fell into the Faraday cup, then measured the electrostatic capacity by the digital charge meter. And use the accumulated charge of unit mass of pharmaceutical to represent the amount of static electricity accumulation. According to GJB 772A-97 explosive test method, 601.3 Impact Sensitivity, type 12 drop hammer apparatus was used to test the impact sensitivity. The special height (H_50_) represents the height from which 2.5 ± 0.002-kg drop hammer will result in an explosive event in 50% of the trials. Test conditions for the dose were 35 ± 1 mg, temperature of 10~35 °C, and relative humidity 80%. The thermal diffusion coefficients of these samples were measured by laser flash method. The sample size is 10 mm × 2 mm (diameter, thickness). The surface of the sample was wiped with ethanol, and the front surface was coated with graphite emulsion with the temperature of 25 °C. The thermal conductivity (*k*) was calculated using the equation (Eq. (1)). Using the detonation wave front of the explosive ionization conductivity, the detonation wave propagation time in the explosive column was measured with a time-measuring instrument and an electric probe. And the detonation velocity was obtained by calculation.

## Results and Discussion

### Microstructure Characteristics

**Fig. 2:**
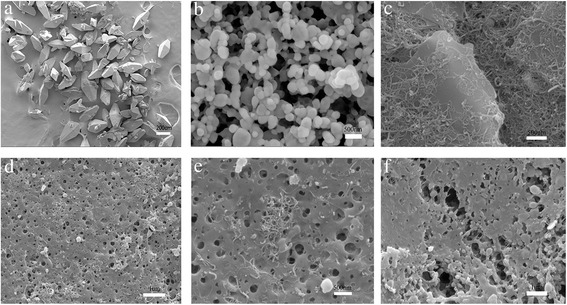
SEM morphologies of CL-20, the mixture of rGO and CNT, and CL-20-based composites: **a** raw CL-20; **b** milled CL-20; **c** rGO + CNT; **d**, **e** CL-20/CNT; and **f** CL-20/rGO + CNT

Figure 1 showed the SEM morphologies of CL-20, the mixture of rGO and CNT, and CL-20-based composites. As we can see, most of raw CL-20 particles were spindle with particle size of about 300 μm (Fig. 2a), and after ball milled, CL-20 particle size was substantially reduced, just about 200 nm (Fig. 2b). As shown in Fig. 2c, the average size of rGO with five layers was 2 μm, and CNT adhered to the rGO and formed complex structure with CNT bridging adjacent rGO. After coated with carbon-based nanomaterials, it was observed that CNT agglomerated in the composites (Fig. 2d, e), which seriously affected the performance of high thermal conductivity. And as shown in Fig. 2f, CNT and rGO were not detected in the samples coated with the mixture of CNT and rGO, indicating that both of them were dispersed evenly and it might also be due to the little amount of them.

**Fig. 3:**
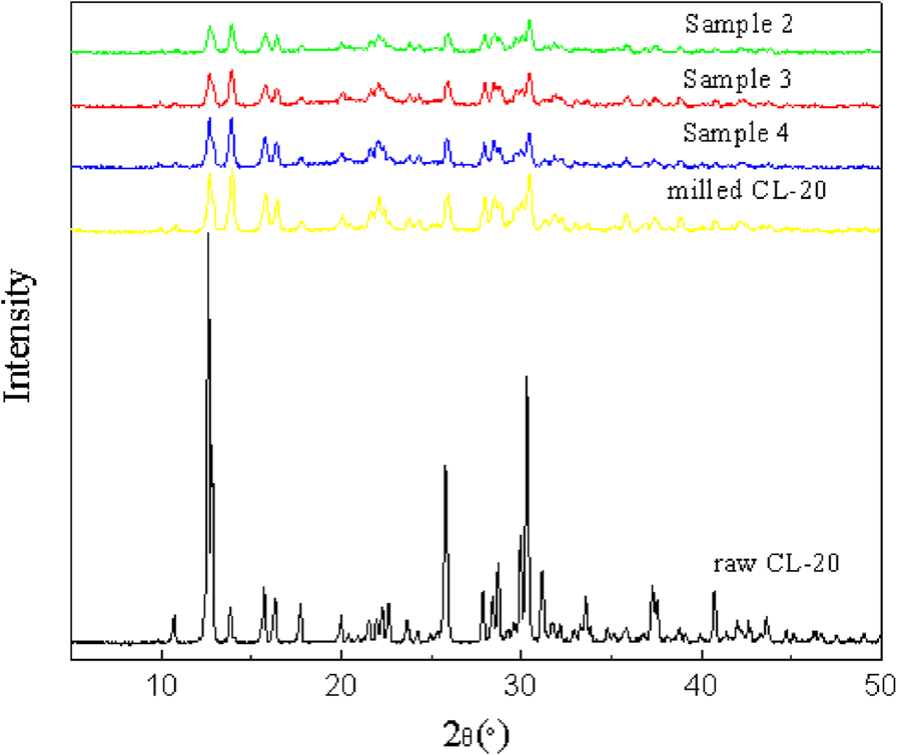
X-ray diffraction patterns of samples

As shown in Fig. 3, there are characteristic peaks at 2*θ* = 12.59^o^, 13.82^o^, 30.29^o^, which is in accordance with the standard ε-form pattern, indicating that the raw CL-20 acquired is ε-form [6, 19]. And the position of the diffraction peaks of the coated samples are basically the same as the positions of the raw CL-20, which indicated that the samples after coating still maintained ε-form [18]. However, at the same diffraction angle, the coated samples correspond to the intensity of the diffraction peaks are significantly weaker than that of the raw material, and the diffraction peaks are partially broadened, which is mainly due to the influence of the particle size of the coating materials.

### Thermal Analysis

**Fig. 4:**
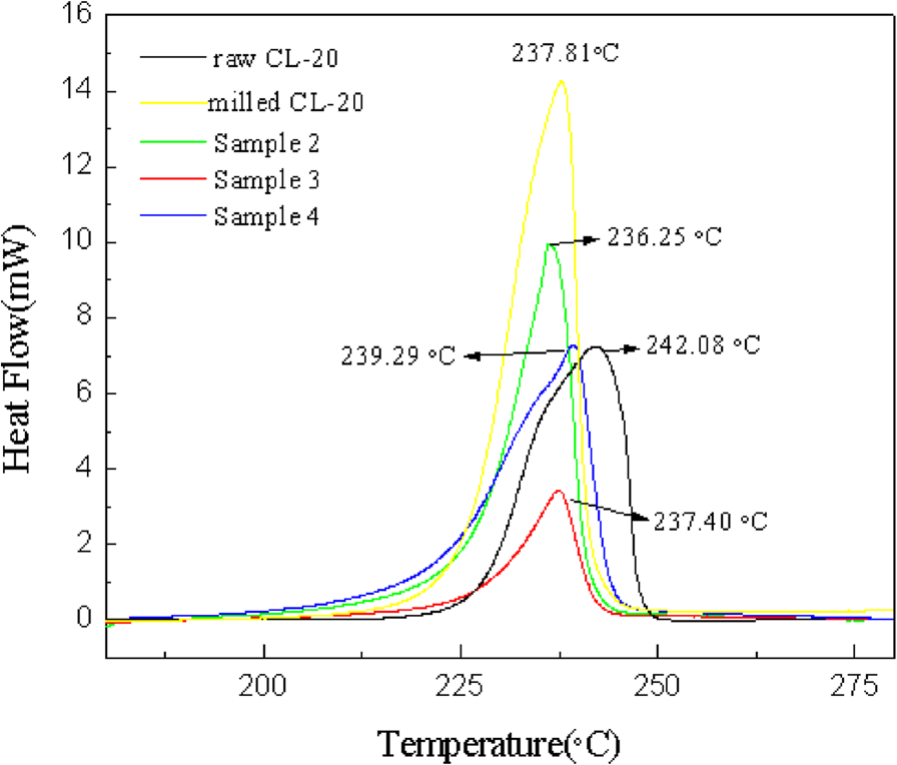
DSC curves of samples

DSC is used to test the thermal decomposition performance of the samples. Shown in Fig. 4 is the DSC curves of the samples with the heating rate of 5 °C/min. The exothermic peak of CL-20 reached the peak point at 242 °C and then dropped sharply, which was consistent with the thermal decomposition of explosives [20]. The thermal decomposition of the coated samples can also be seen from the Fig. 4, and the trend is roughly similar to raw material, and the difference of peak decomposition temperatures between the samples coated with the mixture of rGO and CNT and raw CL-20 is close to 2 °C, which indicated that their compatibility effect is superior than others [21], and the reasons for poor compatibility to others are mostly affected by agglomeration or VDW’ forces. However, at the same heating rate, the decomposition peaks of the coated samples are earlier than those of the raw material, indicating that the composite thermal decomposition reaction was advanced, the rGO and CNT can catalyze the decomposition of CL-20. It can also make explosive molecules decompose easier and more active and also decreased the maximum decomposition peak temperature. Besides, the addition of CNT significantly reduced the enthalpy of explosion decomposition from − 2384.95 to − 779.82 J/g, which might lead to the energy performance of explosives (explosion heat and explosion temperature) weakened in practical applications. Therefore, using rGO which has better thermal stability balances the decomposition enthalpy of the mixture and makes it stabled at − 1897.80 J/g [6]. Besides, the content of CNT in explosive system should also be strictly controlled.

### Sensitivity Analysis

**Fig. 5:**
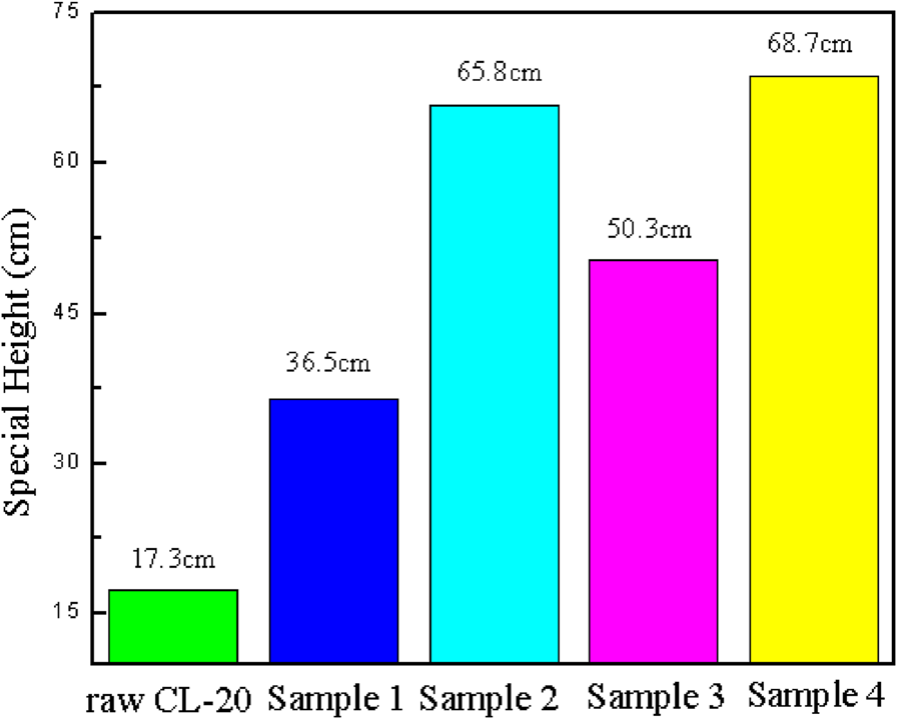
Impact sensitivity of samples

On normal circumstances, special height reflects the sensitivity of explosives, the higher the special height, the more insensitive of explosives, and the higher of safety. As shown in the Fig. 5, the special height (H_50_) of raw CL-20 is 17.3 cm. The special height of sample 2, sample 3, and sample 4 changed from 17.3 to 65.8, 50.3, and 68.7 cm; the impact sensitivity was significantly reduced. This is mainly because, on the one hand, rGO and CNT form a dense protective film on the surface of the CL-20 under the action of a binder, so as to passivate the surface and hardly form the “hot spot” under the external mechanical stimulation. On the other hand, due to the excellent thermal property of rGO and CNT, especially the mixture of them, it is beneficial to heat evenly [18, 22] and reduce the impact sensitivity of the whole explosive system.

**Fig. 6:**
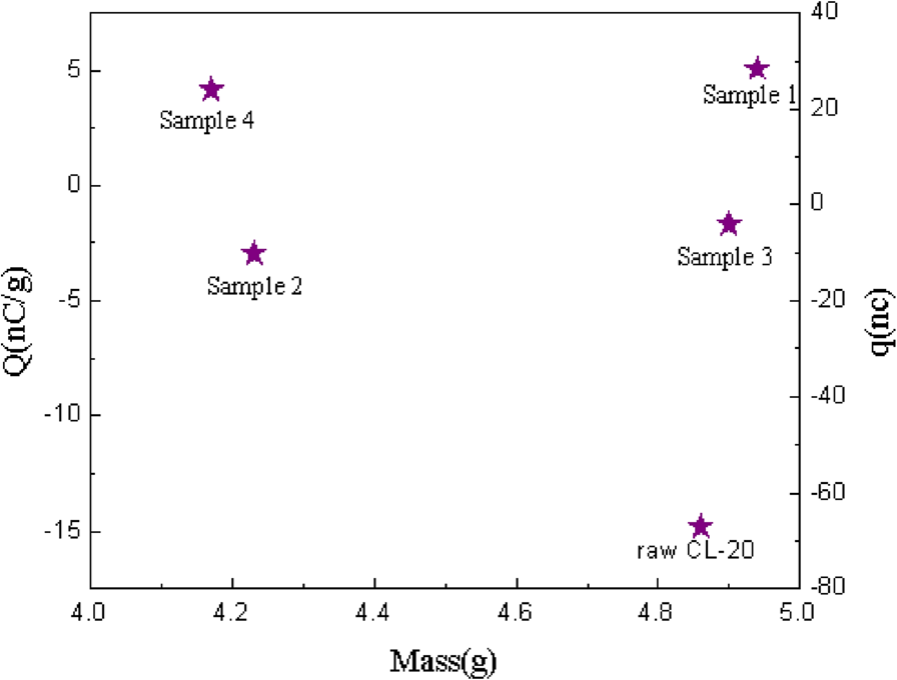
Static electricity accumulation of samples

Furthermore, the amount of static electricity accumulation is an important parameter for evaluating the electrostatic properties of energetic materials and the safety in the electrostatic environment. The static electricity accumulation amount of raw CL-20 and the coated samples are shown in Fig. 6. The static electricity accumulation of the coated samples were significantly lower than that of the raw material, mainly because the CL-20 crystal was bound by the binder and coating materials to a larger particles, reducing the friction during the contact area, thus reducing the friction accumulated charge [23, 24]. What's more, the electrostatic accumulation of CL-20 coated with rGO and CNT’s mixture is mainly affected by the CNT [25].

### Thermal Conductivity Analysis

The thermal diffusivity and thermal conductivity of all samples are shown in the Table 1. It can be found that at 25 °C, the thermal conductivity of raw CL-20 was only 0.143 W/m K. After coating with 1 wt% carbon nanomaterials, the thermal diffusivity and thermal conductivity were significantly increased, of which the samples coated with the mixture of CNT and rGO had the highest thermal conductivity of 0.64 W/m K, and which is 4.5 times of the raw CL-20. This is mainly because both rGO and CNT have very high thermal conductivity, and their application of explosives can significantly improve the thermal conductivity of explosives molecules. In addition, according to the literature, only a very small amount of carbon nanomaterials (rGO or CNT) added to the explosives can achieve significant improvement in effective thermal conductivity [7]. Therefore, in order to achieve the best effect, only 1 wt% of coating material was added in this experiment.

**Table 1:** Thermal conductivity of samples

Samples	Milled CL-20	Sample 1	Sample 2	Sample 3	Sample 4
α(cm^2^/s)	0.00090	0.00123	0.00242	0.00221	0.00251
C_p_[J/(g·k)]	1.111	1.020	1.135	1.035	1.379
q(g/cm^3^)	1.430	1.870	1.860	1.840	1.850
k[W/(m·K)]	0.143	0.235	0.511	0.421	0.640

**Fig. 7:**
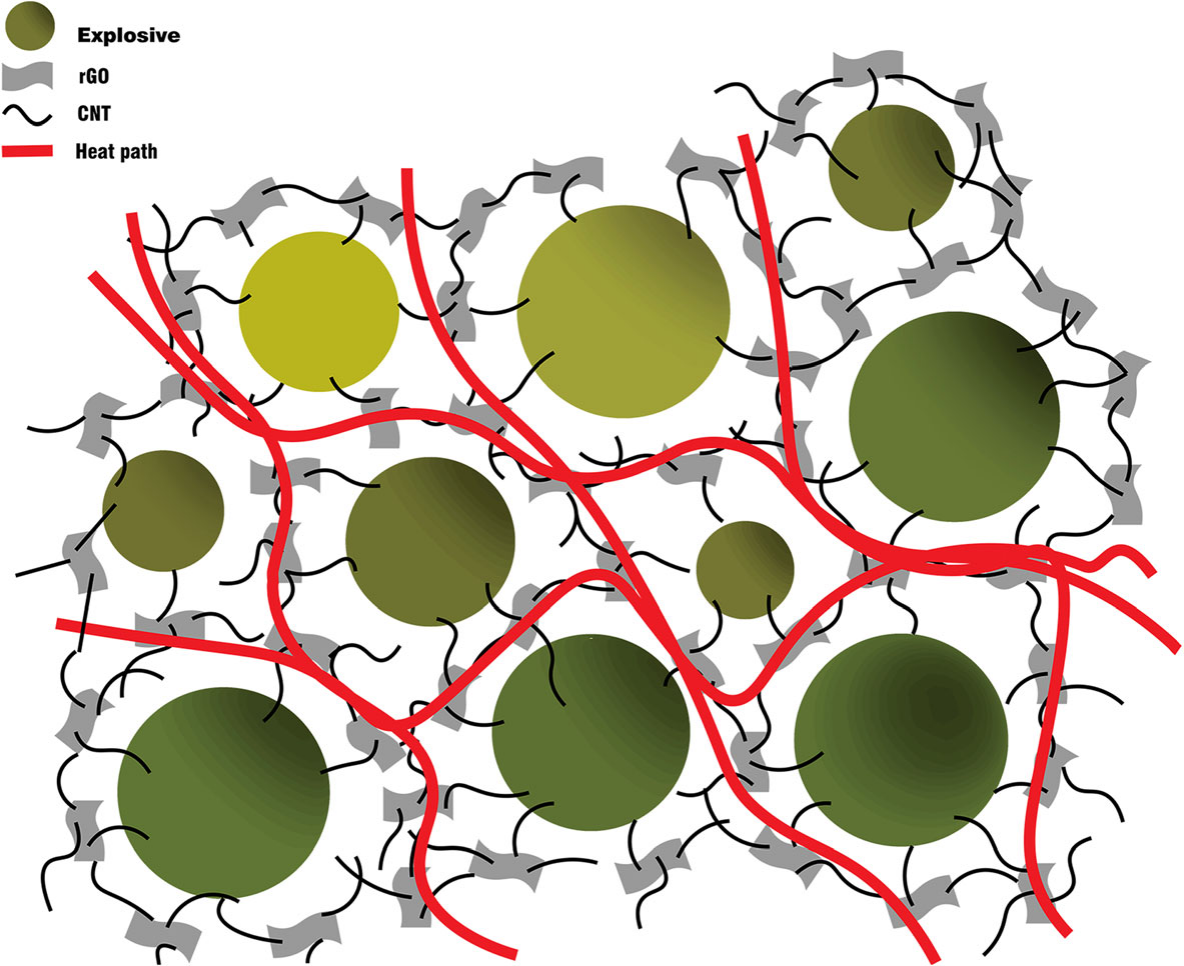
Schematic diagram of thermal transfer of CL-20/rGO + CNT

According to the above mentioned thermal analysis, it can be seen that the mixture of rGO and CNT was more effective for improving the thermal conductivity of CL-20 than the using of rGO or CNT alone. In order to better explore the influence of carbon-based materials on the thermal conductivity of CL-20, simply draw the above mechanism picture. As shown in Fig. 7 (the bottle green sphere represents CL-20 particles, the gray rectangle represents two-dimensional rGO, the black line represents CNT, the red line represents the thermal conduction path, and the blank space represents estane), rGO and CNT have a synergistic effect on improving the thermal conductivity of CL-20. On the one hand, CNT bridged the adjacent rGO and CL-20 explosive particles, and CNT played the role of bridging, which benefit from the better flexibility of CNT [26]. In addition, one-dimensional CNT can provide additional channels for the heat flow of the polymer matrix. And on the other hand, the use of two-dimensional graphene flake’s structure can create more junction points to CNT, which attributed to the high specific surface area of rGO [27]. Since the interaction between the rGO and CNT, it creates more heat conduction paths and provides more paths for phonon transmission, thus forming a three-dimensional network structure of thermal conduction. In addition, due to the high specific surface area of rGO and CNT, it is beneficial to increase the contact area between the coating materials and the explosive matrix and reduce the inter-layer thermal resistance. Besides, rGO has a similar chemical structure with the CNT, so their inter-facial thermal resistance can be significantly reduced [28], thereby enhancing the heat transfer efficiency of the whole system. While for the CL-20, which uses rGO or CNT respectively as the thermal conductive fillers, though both of them have very high thermal conductivity, the interface boundary and defect scattering of CNT can increase the thermal resistance between layers, and the VDW between the rGO also increase the thermal resistance, thereby reducing the overall heat transfer efficiency.

As we all know, in the explosive charges, tiny pores or voids are subject to adiabatic compression, which leads to rapid temperature rise in the pores. When the temperature exceeds the critical temperature, a “hot spot” is formed, heating nearby explosive particles and causing them to decompose to release more heat to cause explosion [29]. In order to reduce the generation of “hot spot,” it is necessary to control the hot spot temperature and heat content, while the high thermal conductivity of the filler materials can effectively reduce the “hot spot” temperature and heat content. Because of its high thermal conductivity and soft properties, rGO and CNT are added into the CL-20 as the fillers, which can not only form a thin coating on the surface of the explosive, complement the voids, and weaken the friction between the particles, but also help the particles heat evenly and quickly spread to reduce heat content. Especially the mixture of them, they can form three-dimensional thermal network to improve the heat transfer more efficiently, just as discussed above. When the “hot spot” decrease, the explosive particles are uniformly heated and not easily affected by external stimulus, thereby reducing the impact sensitivity of the explosive system and ensuring the stability of the explosive. Therefore, it is important to improve the thermal conductivity of the whole system to reduce the sensitivity.

**Fig. 8:**
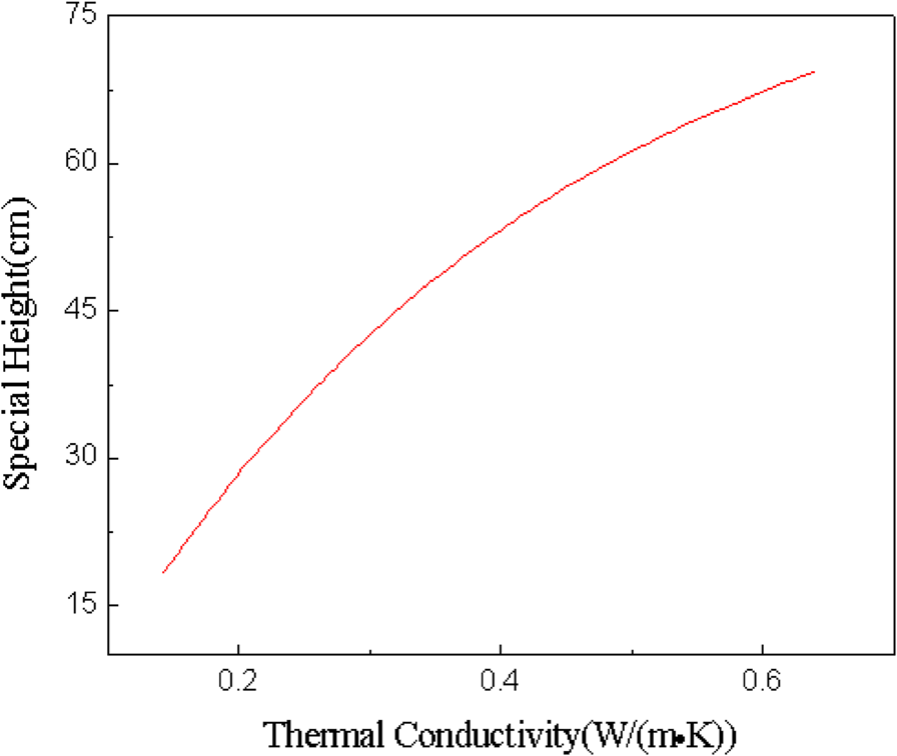
Relation diagram between thermal conductivity and special height

Furthermore, in this study, we performed a linear fit of the thermal conductivity and the special height of the coated samples. As shown in Fig. 8, the relationship between them was positively correlated. As the thermal conductivity of the sample increased, the special height gradually improved, indicating that the sensitivity of the explosive system had been significantly reduced. The result proved that the thermal conductivity of the system did have important influence on the impact sensitivity of Cl-20. What's more, we got the empirical formula (Eq. (2)):

2 $$ y=85.62527-101.06403\exp \left(-\frac{x}{0.35142}\right) $$

where *x* and *y* are the thermal conductivity [W/(m·K)] and impact sensitivity (cm), respectively. Just as we can see, the thermal conductivity and sensitivity showed a clearly positive relationship, which also means as the thermal conductivity increases, the sensitivity of the explosive can be significantly reduced. It also proves that improving the thermal conductivity of the explosive system by adding carbon nanomaterials as thermal conductive fillers did help to reduce the mechanical sensitivity of explosives.

### Detonation Performances

The theoretical detonation performances (calculated by EXPLO5 program) and actual detonation velocity for the raw CL-20 and coated samples are shown in the Table 2 (Theoretical detonation performances of sample 3 and sample 4 both used the theoretical detonation velocities of the sample 1. And since the actual detonation velocity of the raw material cannot be measured, the theoretical value is used for comparison). It can be seen from the above table that the actual detonation velocity of the samples were generally lower than the theoretical value, which may be affected by the ambient temperature, the explosive mixture, the testing instrument, and other objective factors [30, 31]. And as we can see, the detonation velocity of sample 3 reduced 200 m/s than other coated samples, indicating that CNT had a significantly effect on detonation performance, which was consistent with the conclusion of thermal analysis. But the performance of sample 4 changed little, indicating that the different carbon-coated materials used in conjunction have little effect on the detonation velocity of the samples. Although the detonation velocity is weaker than that of the CL-20 raw materials, the overall system is still with a wonderful energy property.

**Table 2:** Detonation performances of samples

Samples	Density (g/cm^3^)	Pressure (GPa)	Temperature (K)	Theoretical velocity (m/s)	Actual velocity (m/s)
Raw CL-20	2.038	44.663	4097.97	9762	–
Sample 1	1.967	40.130	3930.04	9325	8838
Sample 2	1.981	40.667	3932.18	9370	8735
Sample 3	1.981	40.667	3932.18	9370	8472
Sample 4	1.981	40.667	3932.18	9370	8715

## Conclusions

In summary, CL-20-based composites with rGO and CNT did help to increase the thermal conductivity of the explosive system. The fitted formula and curve proved that the improvement of thermal conductivity has a great influence on the sensitivity of the explosive system, and the impact sensitivity of the coated samples were effectively reduced due to the increase of thermal conductivity. In addition, the addition of carbon materials had little influence on the energy of the explosive system. Finally, there are still some shortcomings in this study, such as the effect of different ratios of rGO and CNT on the experimental results had not been considered, so this part will be further explored in the following work.

## Additional file

Additional file 1: Experimental details and equations. (DOCX 20 kb)

## Abbreviations

CFRP: Carbon fiber reinforced plastic
CL-20: 2,4,6,8,10,12-Hexanitro-2,4,6,8,10,12-hexaazaisowurtzitane
CNT: Carbon nanotube
DSC: Differential scanning calorimeter
GNP: Graphene nanoplatelets
H_50_: Special height
HMX: 1,3,5,7-Teranitro-1,3,5,7-tetrazocine
PBX: Polymer-bonded explosive
RDX: Hexahydro-1,3,5-trinitro-1,3,5-triazine
rGO: Graphene
SEM: Scanning electronic microscopy
SWNT: Single-walled carbon nanotube
VDW: The van der Waals’ force
XRD: X-ray diffraction
