# Supplementary material for: Reduce the Sensitivity of CL-20 by Improving Thermal Conductivity Through Carbon Nanomaterials
Source: Nanoscale Res Lett. 2018 Mar 27;13:85. doi: 10.1186/s11671-018-2496-3 (PMC5873462; doi:10.1186/s11671-018-2496-3)
Supplement: Supplementary file 1 — Experimental details and equations. (DOCX 20 kb) [file 11671_2018_2496_MOESM1_ESM.docx]

**Additional file 1**

**Reduce the Sensitivity of CL-20 by Improving Thermal Conductivity through Carbon Nanomaterials**

Shuang Wang ^1^, Chongwei An ^1,2,*^, Jingyu Wang ^1,2*^ and Baoyun Ye ^1,2^

^1^ School of Environment and Safety Engineering, North University of China, Taiyuan, Shanxi, 030051, China; [963623864@qq.com](mailto:963623864@qq.com)

^2^ Shanxi Engineering Technology Research Center for Ultrafine Powder, North University of China, 030051 Taiyuan, China

*** Corresponding authors**, Email address: [anchongwei@yeah.net](mailto:anchongwei@yeah.net) (Chong Wei An)

**Experimental Details**

1. **Preparation of Submicron CL-20**

The particle size, size distribution and morphology of the explosives are essential physical characteristics that significantly influence their sensitivities. Explosives with a small particle size, narrow size distribution and rounded morphology exhibit markedly lowered initiation sensitivity and reduced critical diameter [1]. Mechanical ball milling is a desirable choice, because it is suitable for massive and continuous preparation of uniform morphology crystals, which need to maintain the original crystal form. Herein, the method of mechanical ball milling was used to prepare submicron CL-20. Mainly including: 10 g raw CL-20, 100 ml de-ionized and 200 g zirconia balls were added to ceramic pot fixed in a planetary ball mill. The rotation speed of the planet carrier was 300 RPM. After 180 min, the ground powder was used for sonication to completely remove the zirconia balls, and the milled CL-20 was obtained.

1. **Equations**

 (1)

where k, α, q, and C_p_ are the thermal conductivity [W/(m·K)] , thermal diffusivity (cm^2^/s), density (g/cm^3^), and specific heat capacity [J/(g·K)] of the composite, respectively [2].

References

1. B.Y. Ye, C.W. An, Y.R. Zhang, C.K. Song, X.H. Geng and J.Y. Wang, Nanoscale Res Lett.,2018
2. H. Im and J. Kim, Carbon., 2012, 50, 15
